# Assessing the knowledge, attitudes and practices regarding cholera preparedness and prevention in Ga-Mampuru village, Limpopo, South Africa

**DOI:** 10.4102/jamba.v8i2.164

**Published:** 2016-01-13

**Authors:** Alice Ncube, Andries J. Jordaan, Beverly M. Mabela

**Affiliations:** 1 Disaster Management Training and Education Centre for Africa (UFS-DiMTEC), University of Free State, South Africa

## Abstract

The study assessed the knowledge, attitudes and practices of cholera prevention and preparedness in Ga-Mampuru village (Limpopo, South Africa). Interviewers collected data using a two-pronged method, namely a household questionnaire (open- and closed-ended questions) to assess knowledge and attitudes about cholera and observations to assess practices in the prevention and management of the disease. Additionally, interviewers took pictures with the respondents’ permission. Ninety-six respondents were interviewed. Most respondents (86%) indicated they knew how cholera was contracted with 84% indicating contaminated water as a source. Ninety percent of the respondents indicated they knew how to prevent contracting cholera. All respondents generally knew that cholera could be treated with medicine received at a health-care facility or worker. Fewer respondents (58%) had specific knowledge such as the use of rehydration solutions. The respondents’ high level of prevention practices could be biased. Interviewers observed that many practices were not adhered to, like not washing hands, not using toilet paper and throwing waste in respondents’ yards. Therefore, the community of Ga-Mampuru had not reached a stage of adequate cholera prevention and preparedness in spite of the fact that they were aware of cholera risks and risk-reduction measures.

## Introduction

Cholera continues to threaten many countries and constitutes a major global public-health problem. The Johns Hopkins and the International Federation of Red Cross and Red Crescent Societies (2008: 317) indicates that, amongst displaced populations, diarrhoeal diseases account for over 50% of the deaths during acute emergency phases. This was witnessed in 1994 where cholera and *Shigella* dysentery caused 85% of the recorded 50 000 deaths after the influx of Rwandan refugees in the DRS, north Kivu. In Haiti, *Vibrio cholerae* was introduced following an earthquake in January 2010, and 3990 deaths were reported.

South Africa was considered to be at risk for cholera outbreaks as early as 1971, and the first case of cholera was diagnosed in 1973 (Mugero & Hoque 2001). Küstner and Du Plessis (1991) also report that there were seven periods of cholera epidemics, designated Cholera I–VII, that occurred in South Africa between October 1980 and July 1987. During this outbreak (1980–1987), a total of 25 251 cases of cholera were bacteriologically proven, resulting in 348 deaths that translate into a case-fatality rate (CFR) of 1.4%. Cholera I occurred in the summer of 1980–1981 with the highest number of cases reported from the Limpopo and Mpumalanga Provinces. The first case of Cholera II was reported on 07 August 1981 in the Sekhukhune District of the Limpopo Province where the Greater Tubatse Municipality is located.

The next cholera epidemic was during 2000–2001, with KwaZulu-Natal accounting for 95% of the country’s cases. At 0.21%, the CFR was lower than the national figure of 0.22%, implying good case management in comparison with the World Health Organization’s (WHO) benchmark of < 1% (World Health Organization 2014). Limpopo Province showed great improvement from 465 cases with two deaths in 2001–2002 down to zero cases in 2003. Sporadic, localised outbreaks of cholera continued within the country over the next decade.

**FIGURE 1:**
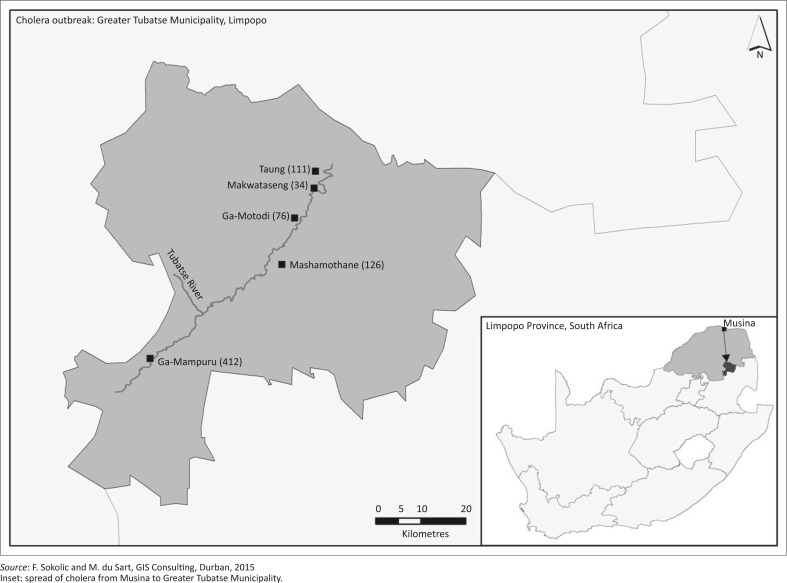
Cholera outbreak in the Greater Tubatse Municipality, 2008–2009.

In 2008–2009, an unexpected serious outbreak suddenly occurred in the northern provinces, originating from Zimbabwe. Limpopo and Mpumalanga were directly affected with the highest CFR of 0.5% in Limpopo, which translated into 26 deaths (UNICEF South Africa 2008). This outbreak, although originating in neighbouring Zimbabwe, brought to the fore the question of cholera management and prevention in South Africa, particularly in Limpopo where no cases had been reported for more than 5 years. During the 2008–2009 outbreak, the first South African case of cholera was registered on 15 November 2008 in Musina, a few kilometres from the Zimbabwe border (UNICEF South Africa 2008). The disease then spread to other areas in Limpopo, affecting mostly the Greater Tubatse Municipality, which reported as many as 759 cases of cholera in five of its villages. The worst affected was Ga-Mampuru (412), followed by Mashamothane (126), Taung (111), Ga-Motodi (76) and Makwataseng (34) (Greater Tubatse Municipality 2009; Figure 1).

Cholera is extremely virulent and whilst about 75% of people infected with *Vibrio cholerae* do not develop symptoms, the pathogens stay in their faeces for seven to 14 days and then return into the environment, infecting other individuals (Global Task Force on Cholera Control 2014). Proactive rather than reactive steps are needed to prevent and prepare for cholera in order to manage the disease. South Africa’s National Department of Health (2006) also identifies the proactive approach as the best way to reduce the risk of cholera spreading in the community. The proactive approach saves valuable time as it replaces the need to first complete an outbreak investigation and allows for more rapid implementation of control measures, which means that many lives can be saved. Some of the proactive measures in place are food safety, water and sanitation, hygiene practices and health education (National Department of Health 2006).

The public’s level of knowledge and hygiene practices contributes to the type of proactive measures that are implemented. In Dar es Salaam, the hygiene practices of the community reflected a lack of knowledge and a negative attitude towards cholera as well as specific misconceptions about the disease (Mpazi & Mnyika 2005). The influence of the measures taken to combat the disease, namely oral cholera vaccination, implemented after the Haiti cholera outbreak was evaluated by Aibana *et al.* (2013). There were fears that the vaccination would reduce recipients’ hygiene practices. Pre- and post-surveys, however, reveal that the vaccination campaign improved the populations’ knowledge of and practices regarding diseases such as cholera. As the levels of knowledge and hygiene practices determine the proactive measures needed to curb the recurrence of cholera, this study assessed the knowledge, attitudes and practices of households in the Ga-Mampuru community regarding cholera preparedness and prevention measures. Consequently, factors were identified that may constrain or enhance such measures.

### The study area

The study area (Figure 1) was limited to Ga-Mampuru (24.761°S 29.877°E), a village located within the Greater Tubatse Municipality in the Limpopo province (South Africa). The researchers chose Ga-Mampuru (population of 7449) because the highest number of cholera cases in the 2008–2009 outbreak was recorded here (Greater Tubatse Municipality 2009; Masombuka 2014).

## Research methodology

This study utilised a two-pronged method to collect the data: (1) a household survey to assess the community’s knowledge of and attitudes about cholera and (2) observations to assess their practices in the prevention and management of the disease.

Three researchers and two assistants formed the research team. The interviewers consisted of one researcher and two assistants; they conducted their observations during the interviews. All researchers acted as monitors and evaluators to determine whether data collection was done according to the study guidelines. Prior to fieldwork, the assistants were comprehensively trained for a day to deliver the questionnaire in order to ensure that the respondents’ responses were a true reflection of their knowledge and not of how data were collected. The training focused on orienting the assistants on the survey objectives, their roles and responsibilities, the general administration of the questionnaires, what to observe, confidentiality procedures and field logistics.

The questionnaire was first piloted (Francis 2007) on five randomly selected villagers in Ga-Mampuru to detect any significant defects in the design and to find out how well the questions were understood by the interviewers and the respondents.

A total of 96 households were randomly selected and considered to be sufficient because the population was homogenous with respect to the variables, namely the type of toilet (pit latrine) and drinking-water source (taps, river). This choice is supported by Maree *et al.* (2007:178) who state that ‘… smaller samples may adequately represent the population in homogenous populations, where members are similar with respect to variables that are important to the study’.

The village is mountainous with a main road and a river dividing it into two sections, each of which has access to the water from the Tubatse River. For data collection purposes, respondents from both sides of the river were interviewed in order to obtain a representative sample. The interviewers conducted the survey in 2011 over a period of 2 weeks.

The household survey questionnaire contained both closed- and open-ended questions, divided into four sections:

- demographic information
- knowledge of prevention of and preparedness for cholera
- attitudes towards prevention and preparedness
- practices relating to cholera prevention and preparedness.

The respondents had to select from pre-determined responses in the close-ended questions that required factual responses whilst open-ended questions allowed respondents to use their own words and express their actual feelings about their knowledge and attitudes towards cholera prevention and management. Multiple responses were accepted from the respondents for certain questions. Bird and Dominey-Howes (2008:45) note that a face-to-face interview is beneficial as complex questions can be explained and clarified, and vague responses can be probed using visual prompts. The respondents were household members who were primary care givers, both women and men, 18 years and older. Non-proportional quota sampling was employed. The interviewers conducted the house-to-house interviews, approaching every second household until the required number was attained. Where nobody was available or willing to participate, the household next-door was approached. Some respondents preferred to complete the questionnaires on their own in their own time, but the interviewers always made sure that each questionnaire was properly completed upon collection. Respondents who could not read were assisted by the interviewers who read the confidentiality statement and purpose as well as the questions and explained where necessary.

At the same time as the survey, interviewers made observations and took pictures (with the respondents’ permission) to identify day-to-day, common practices within the community that could have constrained or enhanced measures intended to prevent or prepare for a cholera outbreak. Observation is a systematic process of recording the behavioural patterns of participants or occurrences without necessarily questioning or communicating with them (Maree *et al.* 2007). Observations can yield information which people are normally unwilling or unable to provide. The interviewers in this study also made observations, which allowed them to identify commonly occurring, day-to-day practices within the community. Interviewers observed the surrounding environment and assessed the hygiene conditions, thereby also familiarising themselves with the physical context in which hygiene practices occurred. This method allowed the interviewers to understand the behaviour and interactions of household members as they went about their everyday activities. Using a checklist and taking pictures, the interviewers also identified hygiene practices that enhanced or constrained cholera transmission. It also included cholera prevention and control practices and preparedness measures, namely:

- the availability, use and cleanliness of toilet facilities
- handling of children’s stools
- washing of hands with soap after using toilets, before preparing food, before eating and after handling children’s stools
- water sources and the treatment of water for home use
- water preservation
- washing kitchen utensils and cutlery
- food handling and waste disposal.

After data collection, the researchers manually checked and coded the quantitative data and entered the data into an Excel spreadsheet for interpretation and analysis. The researchers summarised the qualitative information from the open-ended questions in a word document. Pictures that corroborated discrepancies between respondents’ responses and actual practices are included in this article.

## The sample

The study targeted primary caregivers in the households of Ga-Mampuru village. All of the 96 respondents in the household survey were caregivers, of which 66% were women and 34% men. The respondents’ demographics and sources of cholera information are summarised in Table 1. The majority of the respondents were women aged 60 years and older who had been living in the area for more than 20 years. These respondents had extensive knowledge and experience of the area but had no schooling and were more conversant in their home language, Sepedi.

**TABLE 1:** Respondents’ demographics.

Demographic	Category	Percentage
Age (years)	18–29	19
	30–39	16
	40–49	18
	50–59	18
	60 >	29
Education	No schooling	26
	Preschool	2
	Primary school	10
	Secondary school without Grade 12	22
	Secondary school with Grade 12	20
	Tertiary education	20
Living in Ga-Mampuru village (years)	0–2	0
	3–5	2
	6–10	4.2
	11–20	9.4
	20 >	84.4
Household income (ZAR)	No income	6
	< R1000	16
	R1001–R2000	43
	R2001–R3000	8
	R3001–R4000	4
	R4000 >	23

## Empirical findings and discussion

Ninety-five percent of respondents reported that they had heard of ways and methods of preventing cholera. Tierney, Lindell and Perry (2001) report that people who have heard, understood and personalized a risk are much more likely to adopt preparedness measures than those with no knowledge about an impending danger. It was striking that 5% of the respondents had no knowledge of cholera prevention even though Ga-Mampuru village was a high cholera-prevalence area with the last outbreak recorded in 2008–2009. These respondents (5%) were living in the area at the time of the outbreak, were older than 60 years, and all reported to have had no schooling. Thus, the information that was disseminated did not reach them, or they did not understand the communication. More than half (54%) of the respondents received cholera prevention information from health workers, followed by radio broadcasts (44%) and newspapers (36%). No respondents received information from disaster management officials (Table 2).

**TABLE 2:** Respondents’ information sources on cholera prevention.

Source†	Percentage
Health workers	54
Radio	44
Newspaper	36
Television	34
Brochures, posters and other printed material	20
Magazines	17
Teachers	13
Family members, friends, neighbours and colleagues	13
Church	7
Traditional leaders	2
Disaster management officials	0

*n* = 91.

† Multiples responses possible.

Forty percent of the respondents regarded their knowledge of cholera prevention and preparedness as excellent, 29% as good, 20% as average and 11% as poor. Of those who regarded their knowledge as poor, eight respondents were above 60 years, two were between 50 and 59 years, and one was between 18 and 29 years old.

Most respondents (86%) indicated they knew how cholera was contracted. The cholera sources that respondents identified are given in Table 3. The majority of respondents knew that cholera could be contracted by drinking water from contaminated sources (84%) and by drinking water that became contaminated during transportation (50%). Fewer respondents (41%) knew that cholera could be contracted by eating contaminated food or by eating fruit that were not been peeled and washed (29%). The belief that witchcraft could contribute to contracting cholera was also recorded by Hemson *et al.* (2006) when examining the impact of the 2000–2001 cholera outbreak in KwaZulu-Natal.

**TABLE 3:** Respondents’ knowledge on contracting cholera.

Question†	Knowledge of contracting cholera (percentage yes responses)
Drinking water from contaminated source	84
Drinking water that became contaminated during transportation	50
Eating food contaminated during or after preparation	41
Eating fruit that were not peeled or washed	29
Ingesting faeces and vomit	18
Witchcraft	4

*n* = 83.

†, Multiples responses possible.

Ninety percent of respondents indicated that they knew how to prevent contracting cholera. Respondents’ knowledge of preventing contracting cholera is given in Table 4. The majority of the respondents reported boiling water for at least five minutes (98%) and to store water in a clean container (96%) as prevention measures. The use of prayer to prevent contracting cholera is rooted in the religious beliefs of the community. Koenig (2012) mentions that religion or spirituality and health have historically been interrelated with a separation only occurring in developed countries in recent times.

**TABLE 4:** Respondents’ knowledge on how to prevent contracting cholera.

Question†	Prevention measures (% yes responses)
Boiling water for at least five minutes	98
Storing water in a clean container	96
Using clean toilets	85
Washing your hands thoroughly	82
Drinking water only from an uncontaminated source	76
Washing food with uncontaminated water	65
Disposing of human waste	54
Cooking food or reheating it thoroughly	48
Washing household surfaces and utensils with clean water	46
Avoiding uncooked food unless it can be peeled or shelled	37
Eating food while it is still hot	17
Praying	9
Consulting a traditional healer	4

*n* = 86.

†, Multiples responses possible.

Respondents’ knowledge of cholera treatment is shown in Table 5. Even though 95% of respondents knew of cholera and cholera prevention, all respondents knew that cholera could be treated with specific medicine received at a clinic or hospital or given by a health worker. This discrepancy may be because respondents have a general knowledge of treating illness using appropriate medications. Fewer respondents (58%) had specific knowledge such as the use of rehydration solutions for cholera treatment. All respondents also indicated that they would go to a health facility if they thought they or a family member had contracted cholera.

**TABLE 5:** Respondents’ knowledge of cholera treatment.

Question†	Prevention measures (% yes responses)
Specific medicine given by clinic, hospital or health worker	100
Herbal remedies	94
Homemade oral rehydration solution	58
Prayer	2
Traditional medicines	1
Home rest without remedies	0

*n* = 96.

†, Multiples responses possible.

### Attitudes towards prevention and preparedness

All respondents thought that it was very important to follow the methods for preventing and being prepared for cholera as a way of living in their households. Again, this might reflect a general attitude towards illness prevention and might not be related specifically to cholera. All respondents indicated that anybody could be infected with cholera. Most respondents indicated that the community (94%) and each individual (92%) were responsible for cholera prevention and preparedness.

### The availability of amenities and goods and hygiene practices

The availability of amenities and goods and personal hygiene practices as reported by respondents is given in Table 6.

**TABLE 6:** The availability of amenities and goods and personal hygiene practices of respondents.

Amenities, goods, personal hygiene practices	Percentage (%)
**Water availability†**	
Communal tap	75
Private tap	27
River	23
Rain	2
**Water-storage containers**	
Both narrow- and wide-mouthed containers	68
Wide-mouthed containers	17
Narrow-mouthed containers	15
**Water-treatment methods†**	
Chlorination	35
Boiling	33
Cloth filtration	31
Simple sand filtration	3
Sedimentation	0
Sun exposure	0
**Ablutions**	
Toilet	93
**Goods†**	
Toilet paper	77
Soap for hand-washing purposes	71
Household chlorine bleach	61
Plastic garbage bags	25
Disinfectant	20
Scooper for pet waste	18
**Food safety practices†**	
Wash food with safe water	98
Wash utensils with clean water	90
Cook food or reheat it thoroughly	68
Avoid uncooked food unless it can be peeled or shelled	61
Eat food while it is still hot	52
Not eat uncovered food	52
**Hygiene practices†**	
Wash hands with soap before handling or preparing food	98
Wash hands with soap before eating food	97
Wash hands with soap after using the toilet	95
Wash hands with soap before feeding children	92
Wash hand with soap after attending a funeral	90
Wash hands after changing a diaper or cleaning a child who has gone to the bathroom	85
Defecate in toilet	83
Dispose of human waste promptly	75
Dispose of rubbish and covering it	72
Wash hands with soap before treating a wound	70
Wash hands with soap after handling uncooked food	70
Wash hands after handling garbage	65
Wash hands after blowing nose or coughing or sneezing	57

*n* = 96.

†, Multiples responses possible.

The availability of amenities and goods play an important role in cholera prevention (Funke *et al.* 2009). Only 27% of respondents had running water in their homes. The Institute of Medicine, (US) Forum on Microbial Threats (2009) states that areas with a lack of clean water and adequate sanitation are most at risk of the disease. However, the extent to which good hygiene practices are maintained depends on the availability of certain resources like safe water, which to a large extent also depend on the location and on levels of service delivery. A lack of safe water services such as dry tapes and inadequate service delivery predisposes communities to contamination risks due to the consumption of unsafe water from rivers and other unsafe sources. Some of the respondents (75%) in this study used communal taps, but all indicated that the taps did not always have running water and were therefore unreliable. As a result, some respondents rely on water from a river (23%) or on rainwater (2%). Water is not always stored in narrow-mouthed containers, which minimizes the risk of contamination. No respondent treated their water with sedimentation or exposure to the sun whilst most (35%) treated their water through chlorination.

Although most respondents (93%) had private toilets, this was concerning because 7% of respondents were without toilets and had to use alternative methods to dispose of faeces and urine, the main host of the cholera bacteria. Most indicated that they washed food (98%) and utensils (90%) with safe water. Only 25% of respondents made use of plastic garbage bags for their garbage disposal. Generally, the respondents indicated good personal hygiene practices, especially with food preparation.

### Perceptions of respondents about the cholera outbreak in Ga-Mampuru

The interviewers asked the respondents if they were aware of the cholera outbreaks of 2008–2009. Most (87%) indicated that they were aware of the outbreak. Of these, 73% made further comments. Five respondents indicated that they heard about the cholera outbreak but never believed that it was cholera. Respondents who mentioned that there was no cholera in the village were all men. A total of 66% of those who made comments had similar concerns, which included the following: respondents strongly believed that the municipality had not done much to ensure that the lives of the community members were made easier by providing sufficient resources so that proper prevention measures could be implemented; the respondents emphasized that the community had to revert to strike action to induce the municipality to install taps in certain parts of the village; these taps were subsequently deemed not sufficient because they were too far away for some of the villagers and most of the time did not have running water; and they commended health workers’ efforts in educating the community on cholera prevention and preparedness some respondents’ (18%) comments were not relevant to the topic whilst 16% had a different view. Some of their comments are given below and include perceptions that the water was contaminated with poison and that the river was not the source of cholera. Comments also include a positive view on the government’s cholera prevention activities and scepticism about whether it was cholera that killed members of the community.

Participant 11, a man aged between 30 and 39 years, with a secondary level of education, but not grade 12, said:

‘There was never cholera outbreak in our village in 2008–2009. The government officials lied about cholera outbreak during that time. The truth about that incident is the fact that it was a poisonous chemical because it also killed animals. Cholera cannot kill a person in a second or two days after drinking water. Therefore there was no cholera in our village because we have been drinking water from the river.’

Participant 15, a man aged 60 years or more, with no formal schooling and who had been living in the area for more than 20 years, said:

‘But I do not think it was cholera because we have been living in this village many years drinking river water but we are still healthy.’

Participant 16, a man aged between 40 and 49 years, with grade 12 and who had been living in the area for more than 20 years, said:

‘There was no cholera in our village; it was a poison in our canal water. Somebody washed poisonous container inside it.’

Participant 38, a woman, aged between 40 and 49 years, said:

‘I do salute the South African government for the best role they have played about prevention of cholera disaster in our village.’

Participant 52, a man aged 60 years or more, with no formal schooling and who had been living in the area for more than 20 years, said:

‘We have been living in this village for many years drinking water from the river and mountain stream, if it was cholera people were supposed to be dying every year but we are still living a healthy life.’

Participant 74, a man aged between 50 and 59 years, with a secondary level of education but not grade 12 and who had been living in the area for more than 20 years, said:

‘I heard there was cholera but I do not think it was cholera because cholera cannot kill people in one day we lost many people in a week.’

### Observed actions in community

The results highlighted a high level of cholera awareness in the community, but responses could be biased in favour of desirable actions for reducing the risk for cholera. The interviewers’ personal inspections confirmed the suspicion of biased responses. Social-desirability bias might account for the respondents’ overreporting on desirable actions for risk-reduction concerning cholera. These are explained and summarised in Table 7. Even though the households were not earning large salaries, with 43% reporting an income of between R1001 and R2000, they all possessed a positive attitude and believed that it was their responsibility to follow cholera prevention and preparedness measures. However, it became clear that, due to their household income, they were forced to prioritise their needs, thus neglegting certain practices such as washing hands without soap or washing dishes in dirty water without a detergent.

**TABLE 7:** Summary of interviewers’ general observations.

Practices	Observation
The availability and use of toilets	Seven participants indicated that they did not have toilets, and the interviewers confirmed that, of the seven, five toilets were full and could not be used anymore (Figure 2), one toilet was opened and full, and one person had no toilet in the yard. Most participants who claimed to use toilet paper put newspapers and paper books in their toilets instead of toilet paper whilst some had nothing at all.
Hand-washing practices	Many respondents were observed not washing hands beforeeating in spite of reporting the contrary.Many children did not wash hands after using the toilet.
Water preservation or storage	Children drank water directly from big containers and used the same jug to fetch water.
Washing kitchen dishes	Dishes were washed outside of kitchens in dirty water (Figure 3).
Water collection	Some community members were seen collecting water from the river with both wide-opened and narrow-opened containers with their feet inside the river and the hands touching the insides of the containers (Figure 4).
Food handling and waste disposal	All respondents had waste thrown in their yards, a number included dirty baby diapers (Figure 5).

**FIGURE 2:**
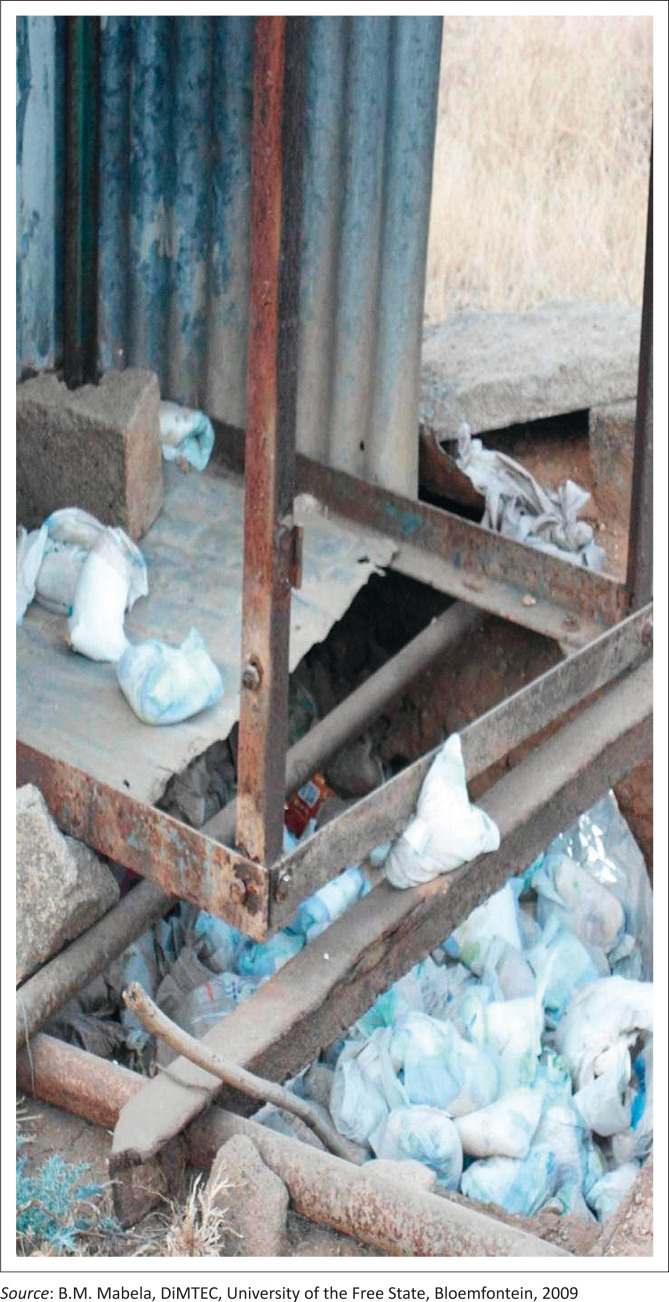
An open toilet filled with soiled nappies.

**FIGURE 3:**
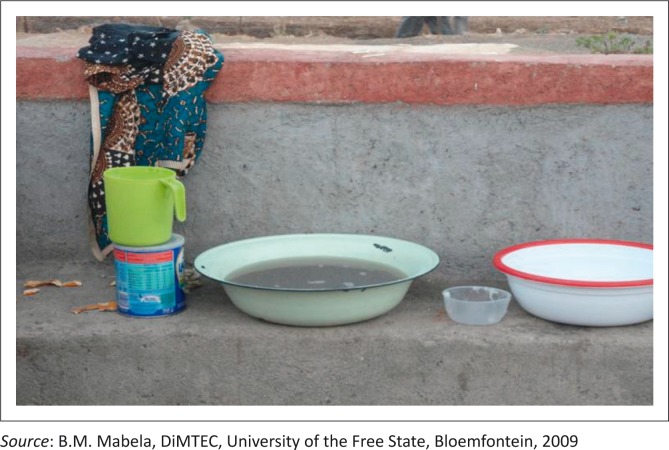
Dirty water used for washing dishes.

**FIGURE 4:**
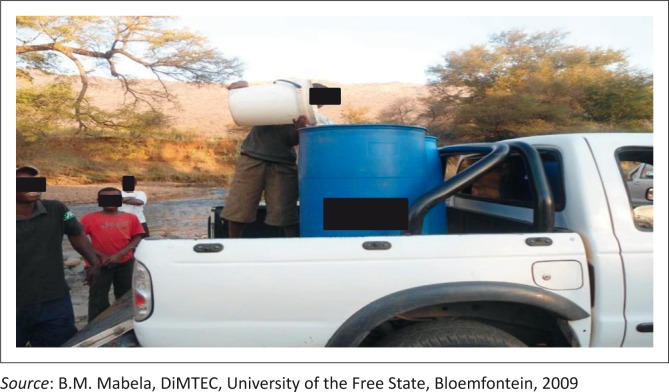
People fetching water from Tubatse River.

**FIGURE 5:**
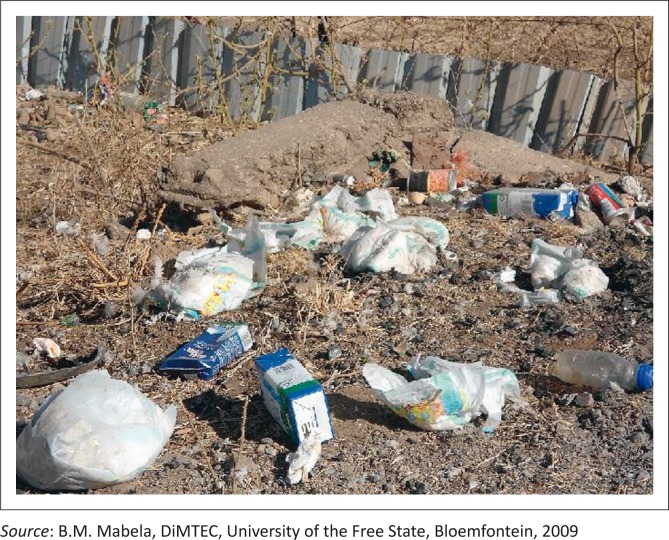
A dumping area in the backyard of a respondent.

## Recommendation

In line with the findings of this study, the following recommendations are made to ensure the implementation of effective and efficient measures for the prevention of and preparedness for cholera in Ga-Mampuru village:

- The relevant departments or organisations should consider conducting coordinated periodic awareness programs to bridge the gap between the knowledge and practice of the Ga-Mampuru community. The awareness programs should aim at disseminating information to make people aware of cholera in the language that they clearly understand, which is Sepedi, and it should use a method that reaches a wide range of audiences such as the print media, radio and television. These programs should be mainstreamed in the current community-development programs that involve schools, clinics, churches and community forums. The community must be encouraged to take responsibility and an interest in building their resilience. In addition, it is recommended that the municipality establish a community-information centre that can serve as an administration facility for all affairs of the community, including documentation of records for community programs and preserving information that can be easily accessed by all members of the community.
- During the study, it was noted that the majority of the elderly respondents, those aged 60 years and above, were illiterate, and hence it is recommended that house-to-house visitation and physical demonstrations would be ideal as a method for raising awareness because the distribution of pamphlets and raising awareness at mass gatherings may be less effective.
- Capacity building should not be limited to professionals and personnel involved in matters of cholera prevention and preparedness but should also focus on building the knowledge, attitude and awareness of the entire community by empowering some of the community members. This can be done by training them in a way that they are able to transfer their knowledge to their fellow community members. In view of this, each community member should be encouraged to be an agent of behavioural change at home.
- Most of the respondents still relied on untreated water from the river for their daily supply whilst some had no or limited access to adequate sanitation facilities. These residents are therefore at high risk and living under the threat of cholera. Insufficient resources restrict them from putting their knowledge, positive attitude and dedication into practice. These households should therefore be identified and encouraged to become involved in diversified sustainable livelihoods and other income-generating activities, which should enable them to provide themselves with essential commodities such as detergent, soap and toilet paper.
- *‘Prevention is better than cure’*. It is therefore of the outmost importance that sufficient funds, specifically intended for matters relating to the prevention and preparedness of cholera, be allocated to the relevant department in the municipality. This would also allow the municipality to conduct training and education, to monitor, evaluate and capacitate officials and to develop and maintain adequate and relevant infrastructure.

## Conclusion

This study assessed the knowledge and attitude as well as the preparedness and prevention practices concerning cholera of respondents in Ga-Mampuru village, a community that was affected by the cholera outbreak in 2008–2009. Most respondents knew how cholera was contracted and how to prevent the contraction of cholera. All respondents knew that cholera could be treated with medicine received at a health-care facility or from a health care worker. The use of a two-pronged approach in the research revealed, however, that respondents’ high level of prevention practices could be biased. Interviewers observed that many practices, which respondents claim to be following, were in fact not followed. These include not washing hands, not using toilet paper and throwing waste in their (respondents’) yards. Therefore, the community of Ga-Mampuru had not reached a stage of adequate cholera prevention and preparedness in spite of the fact that they were aware of cholera risks and risk-reduction measures. They knew about most aspects of cholera prevention and illustrated a positive attitude towards cholera prevention and preparedness, but they do not always put these into practice. It is therefore of utmost importance to embark on massive risk-reduction activities in communities such as Ga-Mampuru village.
